# Understanding and engaging with vocalisations during labour and birth: A qualitative interview study of midwives' experiences and approaches

**DOI:** 10.1016/j.ijnsa.2026.100548

**Published:** 2026-04-30

**Authors:** Laura A. Zinsser, Nancy I. Stone

**Affiliations:** Midwifery Research and Education Unit, Hannover Medical School, Carl-Neuberg-Str. 1, Hannover 30625, Germany

**Keywords:** Midwifery, Childbirth, Singing, Clinical skill, Assessment of health care needs, Qualitative research

## Abstract

**Background:**

Spontaneous vocalisations are a common reaction to pain. During childbirth, birthing women often produce vocalisations that shift in tone and intensity in response to the demands and dynamic process of childbirth. Research has shown that midwives can distinguish between different vocalisations; however, the embodied, experiential, and tacit knowledge underpinning this skill remains largely under-researched.

**Objective:**

To explore and analyse midwives’ experiences and approaches with vocalisations during labour and birth.

**Methods:**

This study employed a constructionist approach within an explorative qualitative design to explore and analyse midwives’ experiences and approaches to vocalisations during labour and birth. The study was conducted throughout Germany. Nineteen midwives with experience as the primary healthcare provider for birthing women in free-standing birth centres, hospital labour wards, or at home births participated in the study. Semi-structured interviews were conducted between October 2024 and February 2025. Data were analysed using reflexive thematic analysis according to Braun and Clarke.

**Results:**

Four themes were generated through iterative engagement with the data. These are: (1) Developing and training auditory skills: learning to listen and attune to birth sounds; (2) Recognising nuances of childbirth sound patterns; (3) Combining childbirth vocalisations, sensory perceptions and embodied cues; and (4) The multi-faceted nature of working with sound. The data showed that midwives developed their auditory skills over time, either through deliberate engagement with vocalisations or with guidance from colleagues and mentors. Integrating birthing women’s vocalisations added depth to midwives’ assessments and provision of care. While several midwives described how they intentionally worked with vocalisations as a component to accompany and support labour, others noted that they rarely made use of vocalisations. When midwives reported that they worked with vocalisations, they described using them to support well-being and physiological processes during childbirth.

**Conclusion:**

Vocalisations are an integral part of labour and birth. The findings highlight that understanding and interpreting these sounds can serve as a meaningful component for supporting well-being, physiology as well as recognising physiological processes and deviations from these during childbirth. Incorporating training in auditory perception into midwifery education would prepare students in their practice-based skills and support non-technological approaches.

**What is already known**

• Midwives and other healthcare professionals working in hospital labour wards can distinguish between vocalisations made in the first and second stage of labour.
• Midwives conceptualise childbirth sounds along a continuum, from sounds associated with coping to those associated with difficulty or distress.

Alt-text: Unlabelled box dummy alt text

**What this paper adds**

• Midwives develop and train their auditory perception through professional experience, along with guidance and feedback from colleagues or mentors.
• Midwives use auditory perception and interpretation of vocalisations as key tools, integrating these with other perceptions and additional forms of assessment.
• Some midwives use situation-dependent vocalisations as a component to support the unique and individual physiological childbirth process.

Alt-text: Unlabelled box dummy alt text

## 1 Introduction

Pain is often expressed through vocalisations, such as moaning, howling, screaming, whimpering, or gasping (Helmer et al., 2020). In many contexts, pain is understood as a symptom of illness or injury, and is therefore typically treated with pain medication for relief (Chou et al., 2020). In nursing and clinical care, additional approaches are used alongside pharmacological pain relief (Hämäliäinen et al., 2022). In the context of acute and chronic pain, non-pharmacological approaches such as performing music, or listening to music, have been shown to support coping with the intensity and quality of pain (Irons et al., 2020; Arnold et al., 2024; Ou et al., 2024). During childbirth, pain takes on a different meaning. Rather than being a sign of pathology, it is recognised by many as a physiological component belonging to the process of giving birth (Whitburn et al., 2019). Birthing women who embrace this approach therefore draw on individual coping strategies to manage contractions without analgesia (Zinsser and Engebakken Flaathen, 2026; Smith et al., 2006;). Many do so by spontaneously or intentionally vocalising, as both an expression of the intensity of contractions and a strategy for dealing with the pain (Zinsser and Engebakken Flaathen, 2026; Revision, 2026; Pierce, 1998). These vocalisations include humming, moaning, producing long and deep tones (vocal toning), and roaring (Zinsser et al., 2025). Thereby, low-pitched, steady sounds are often associated with coping, whereas other sounds may signal distress or difficulties (Roosevelt et al., 2025; McKay and Roberts, 1990; Pierce, 1998).

## 2 Background

### 2.1 Working with birth sounds

In the first stage of labour, the cervix thins (effaces) and dilates to around 10 cm. In the second stage of labour, the baby descends through the vagina to the perineum, after which the baby is born. In each phase of labour, birthing women may produce sounds either as an immediate response to the intensity of contractions or intentionally as a tool to cope with the physical sensations (Zinsser et al., 2025; Leppänen et al., 2018). A small Australian study demonstrated that midwives could distinguish between birthing women’s vocalisations during the first and second stages of labour (Baker and Kenner, 1993), suggesting that midwives hold an internalised understanding of how vocalisations reflect the stage and course of labour. In McKay and Roberts (1990) study, healthcare providers demonstrated that they rely on auditory perception to interpret variations in vocal expressions during the second stage of labour. The authors summarised these perceptions into five categories of sounds, identifying, among others, sounds that signal how well the birthing woman is coping (McKay and Roberts, 1990). Drawing on these categories for the second stage of labour, healthcare providers were able to tailor their care to the individual needs of the birthing woman (McKay and Roberts, 1990). However, the idea of a specific or appropriate tone for vocalisations, which can be perceived by healthcare providers, contrasts with the experience of birthing women, who value a wider range of vocal expressions as a means of coping with childbirth (McKay and Roberts, 1990; Danford et al., 2022; Revision, 2026). At the same time, both midwives and birthing women share the view that screaming is not helpful during labour. Birthing women describe that, when they scream, they either feel afraid or are drawn into a state of fear (McKay and Roberts, 1990; Zinsser et al., 2025). Furthermore, midwives acknowledge that birthing women can experience shame when vocalising during childbirth (Roosevelt et al. 2025; Pereira et al., 2025). Research shows that some healthcare providers feel a responsibility to encourage vocalisation, recognising it as a helpful coping mechanism despite the potential for embarrassment or shame (Roosevelt et al., 2025). As demonstrated in several studies, care providers can either inhibit or facilitate birthing women’s vocal expressions, thereby shaping how these are expressed during labour. Such support to facilitate birthing women’s vocal expressions can be expressed verbally or through embodied interactions from the midwifery care providers (Pereira et al., 2025; Roosevelt et al., 2025; Danford et al., 2022; Zinsser and Stone, 2025). For example, midwifery care providers recognise that birthing women can be supported in finding beneficial vocal expressions, particularly when they begin using discouraging, repetitive phrases (e.g., “no, no, no”) during labour (Roosevelt et al., 2025, p. 4).

### 2.2 Training and awareness on vocalisations in childbirth

Midwives, in particular, acknowledge their role in guiding birthing women toward more constructive, health-oriented vocal expressions and affirmations. Training in this area, however, appears uneven across professional groups (Roosevelt et al., 2025; Pereira et al., 2025). In a qualitative study conducted in the United States, doulas were the only birth companions who reported having specific training to work with vocalisations during childbirth (Roosevelt et al., 2025). In contrast, midwives, nurses, and obstetricians described learning to engage with birthing sounds informally through practice rather than through formal instruction (Roosevelt et al., 2025; Pereira et al., 2025). In addition, studies show that hearing is a skill that can be developed, and that midwives generally have more exposure to vocalisations in their care of birthing women than obstetricians (Baker and Kenner, 1993; Valente et al., 2025a). In one study (Valente et al., 2025a), obstetricians were unable to associate short, clearly identifiable acoustic frequencies with the corresponding phases of labour (audio file: first phase and expulsion phase from Valente et al., 2025b). These differing levels of training and awareness in the educational systems, healthcare structures, along with cultural attitudes toward vocalisation, seem to influence how both birthing women and healthcare providers experience and interpret vocalisations (Danford and Mercein, 2017; Zinsser and Stone, 2025; Navarro-Prado et al., 2022).

## 3 Purpose of the study

Continuous change has characterised midwifery practice across generations, shaped by technical advances and evolving perspectives. As the profession continues to develop, it is essential to recognise and preserve the embodied and tacit knowledge and skills that midwives contribute to the care of birthing women (Stone et al., 2023). However, research exploring how midwives understand and engage with vocalisations during labour and birth is still limited. The aim of this study, therefore, was to examine and analyse *midwives’ experiences and approaches with vocalisations during labour and birth.* Specifically, this research explores how midwives work with sound; how they attune to birthing women’s vocalisations; how they reflect on their own professional responses; how they act intuitively; and how they support physiological processes through the use of vocalisations during childbirth.

## 4 Methods

The methods section was written using Tong et al.’s (2007) criteria for reporting qualitative studies (COREQ).

### 4.1 Theoretical framework

This study employed a constructionist approach within an explorative qualitative design to explore and analyse *midwives’ experiences and approaches to vocalisations during labour and birth*. Constructionism posits that all knowledge, and therefore reality itself, is socially constructed through human practices and interactions. These practices emerge from the dynamic relationship between individuals and the world. Practices are continually shaped, negotiated, and transmitted within social contexts (Cotty, 1998). From this perspective, the meaning of vocalisations in childbirth is not fixed but instead constructed through midwives’ embodied engagement with birthing women and their environments. As Merleau-Ponty (1962) emphasises, perception is an interpretive act. In this sense, hearing is not merely passive but an active process through which meaning is constructed. Thus, auditory perception in midwifery practice involves interpretation and relational understanding rather than simple sensory registration, and becomes evident in the observations midwives make during labour. Constructionism supports an exploration of how midwives’ perspectives, experiences, interactions, and interpretations of vocalisations are socially enacted and passed on within the context of childbirth.

### 4.2 Research participants

Purposive sampling was used to recruit midwives who met specific inclusion criteria: Participants were required to have experience with vocalisations during childbirth and at least two years of professional experience as a primary caregiver accompanying birthing women. Several labour wards across all 16 federal states of Germany were contacted and asked to display study information or circulate it via email to colleagues. In addition to this, the German Association for Quality in Out-of-Hospital Birth (QUAG, 2025) disseminated the study information through their newsletter, thus reaching midwives who provide home births and/or free-standing birth centre care (FSBC). In total, 19 midwives from diverse professional settings (home (*n* = 5), free-standing birth centre (*n* = 7), hospital labour ward (*n* = 7)) were interviewed. Two midwives worked in more than one setting (listed in their primary professional setting). Participants were aged between 28 and 75 years. They lived in the northern (*n* = 2), eastern (*n* = 3), southern (*n* = 6), and western (*n* = 8) regions of Germany and had between 2 and 35 years of experience working as primary healthcare providers for birthing women. At the time of the interview, four participants were either on parental leave, working as an educator, or were retired. Many participants defined the term vocal toning (German: Tönen) as any kind of sound produced by birthing women. Therefore, when midwives did not describe a specific sound in the interview, we used the umbrella term 'vocalisation' in the text.

### 4.3 Setting

This study was conducted across the Federal Republic of Germany. Given the geographical distribution of the participants, interviews were carried out via video conference, telephone, or in person. All interviews were facilitated by the first author.

### 4.4 Data collection

The interview guide for the semi-structed interviews was developed by the first author and refined through collegial discussions to ensure that the questions were open-ended, non-directive, and consistent with the study’s constructionist approach. No pilot test was done. All interviews were recorded using a handheld device and transcribed verbatim. All vocalisations and sounds demonstrated in the interviews were transcribed into the International Phonetic Alphabet (IPA) by a speech-language pathology instructor and clinical linguist to represent the original sounds produced by midwives (International Phonetic Association, 2025). The IPA transcriptions are indicated with a square bracket in the text. Pseudonyms were applied during transcription to protect confidentiality. No field notes were made. The duration of the interviews was between 40 and 131 min. In line with reflexive thematic analysis, interviews were conducted until the dataset was sufficiently rich to address the aims of the study, in line with Braun and Clarke (2022), with attention to capturing a range of experiences and practice settings.

### 4.5 Data analysis

Data were analysed using the six steps of reflexive thematic analysis as outlined by Braun and Clarke (2022). The analytic process began with immersion in the data through repeated reading of the interview transcripts to support familiarisation. LAZ coded all the transcripts inductively, generating codes that captured patterns of meaning relevant to the research aims. Two interviews were independently coded by LAZ and NIS and subsequently discussed to facilitate reflexive dialogue about the coding process and the developing themes. Analysis was conducted iteratively, moving between data, codes, and developing themes to capture both shared and nuanced meanings in the dataset. After initial, potential themes were generated, these were refined and named to represent their analytic essence. Throughout the process, text passages were discussed with peers; these ongoing discussions enhanced reflexivity and ensured coherence between the themes and the full dataset. In the final phase, the findings were written up, utilising participant quotes to illustrate key themes and to preserve the authenticity of the participants’ voices. MAXQDA was used to support data management.

### 4.6 Reflexivity and trustworthiness

The first author conducted the interviews. She is a full-time postdoctoral researcher with 13 years of experience as a free-standing birth centre and home birth midwife. She has a positive attitude regarding vocalisations during childbirth. She was aware that she needed to reflect upon her personal bias throughout the research process. For this purpose, she used journaling and had a pre-understanding interview before commencing data collection. The second author is a researcher and has been practicing midwifery for 24 years in hospital labour wards and in a free-standing birth centre. She encourages vocalising during labour and has taught this in birth preparation courses.

Lincoln and Guba's (1985) criteria for establishing trustworthiness were applied throughout the study. Credibility was enhanced through the researchers’ professional backgrounds in midwifery and qualitative research. Transferability was achieved by recruiting midwives from diverse regions of Germany and across different work settings, ensuring variations in experiences and perspectives. Dependability was achieved through the use of semi-structured interviews that enabled an in-depth exploration of participants’ views and provided a consistent yet flexible data collection process. Confirmability was strengthened through collaborative discussions, double coding of selected transcripts, and reflexive journaling. Authenticity was demonstrated by presenting a range of perspectives that reflect the diversity of midwives’ experiences with vocalisations during childbirth.

### 4.7 Ethical considerations

Hannover Medical School granted ethical approval in February 2024 (Number: 11,270_BO_K_2024). Before the interviews, participants received the information leaflet and gave written consent. To protect the confidentiality of participation, a demographic table will not be provided.

## 5 Results

### 5.1 Theme 1: Developing and training auditory skills: learning to listen and attune to birth sounds

The midwives who took part in this study shared that they had to develop their auditory skills when they first started working with birthing women. While a few midwives stated that they developed these skills during their professional training, most shared that they learned to perceive, understand, and interpret vocalisations through working with birthing women after years of experience. During this learning process, they discovered how to perceive different vocalisations and learned to interpret them with the help of assessment modalities, in order to evaluate labour and provide needs-based care. For some, a colleague or mentor helped them develop their listening skills, drawing their attention to attributes of birthing women’s vocalisations with which they were not yet familiar.

Marta shared that, when working with midwifery students, she noticed that they had not yet developed the skills necessary to understand and interpret birthing women’s vocalisations. Her students were able to carry out actions under instruction but were not yet able to initiate care based on their own perceptions.

But yes, maybe that's what you might call expertise, something you gain with more professional experience, yes. Oh, I think someone very young wouldn’t be able to hear that yet. They can't do that yet [interpret vocalisations] because they haven't had the opportunity to observe many births. When I work with students now, I often watch [them] and think, ‘Hey, this is glaringly obvious, why isn't she doing anything [to help]?’ And then I realise, no, she simply doesn’t hear that yet. It’s all fine, just tell her [what needs to be done] and that's fine. (Hospital midwife Marta)

Some midwives noted that, when they accompanied one birthing woman throughout the whole childbirth process (one-to-one care), this facilitated the development of their auditory skills. Lise had 8 years’ experience and elaborated:

And in the context of having already attended several births. You learn to connect things together, so that you simply notice, yes, the vocal tonings are different now. It could be heading in that direction, let's continue to observe. But if it changes to a certain, different sound, then you say, okay, I should take a look here, because now it doesn't sound like it's still productive. Just connecting these experiences with each other. It's also like how a mother learns to hear what her child is trying to express when it is crying. We know that when a child is hungry, it cries differently than when it has a full diaper or when it feels uncomfortable. And I believe that it is exactly the same learning process for us midwives when we have the opportunity to accompany women holistically during childbirth. (Homebirth midwife Lise)

Hanne developed her auditory skills with the help of colleagues, who shared their perceptions and expertise with her.

I think it's something you learn through experience over the years. And you learn it from the older midwives when they go in and say, ‘Oh’ [because they have heard something], or when you hear them [the birthing woman] outside the closed door [to the labour room], ‘Oh, that sounds difficult’, or, ‘We'd better go and have a look’. (Hospital midwife Hanne)

A few midwives developed their interpretive skills through deliberately listening and attuning to the sounds, rather than merely hearing them. While listening, they engaged in self-reflection, attuning themselves to the sounds and asking critical questions about what they heard. Elfriede, a retired midwife who had 30 years’ experience in supporting women during childbirth, expressed:

Well, midwifery support has always been a lot of inner work for me. First, I have to tune in - What am I experiencing? What thoughts are coming up? - And only then do I compare that with, let's say, with my knowledge of physiology and taking action based on [birth] physiology. (Retired FSBC midwife Elfriede)

The midwives in this study explained that, through experience, they enhanced their sense of hearing, which in turn refined their cognitive capacity and ability to discern and interpret vocalisations during childbirth. This skill evolved over time as they learned to relate what they heard to other perceptual cues and clinical parameters. The process involved constructing meaning from the vocalisations and internalising these interpretations, so that, over time, guidance from others was no longer necessary. Because this skill was often not formally taught, the capacity to hear and interpret childbirth sounds was largely acquired through practice and occasionally supported by colleagues and mentors. A few midwives described working more deeply with vocalisations. They listened attentively, reflected critically, and explored what the sounds revealed about the birthing process.

### 5.2 Theme 2: Recognising nuances of childbirth sound patterns

While only a few midwives described engaging in focused listening, most shared being able to recognise broad patterns and nuances in childbirth vocalisations. Most said, based on their experiential assessment, that they believed they could hear the stage of labour, the dynamics of the childbirth process, and emotional distress, including fear. A few midwives described being able to use women’s vocalisations to interpret when labour deviated from a healthy, physiological labour. For example, they described that when they heard a quality of pain in the vocalisations that appeared disproportionate to the expected intensity of physiological labour, they made the assessment that the cervix could be rigid or spasm. Some spoke of hearing fetal malposition based on the birthing women’s vocalisations. Midwives also described hearing indicators of healthy progress, including physical relaxation, the birthing women’s mental and physical strength, and their acceptance of the birthing process. Further, some participating midwives also perceived embodied phenomena, such as the pattern of contractions, the effectiveness of contractions, the baby’s descent into the pelvis, and the onset of crowning, through changes in vocalisation and sound patterns.

Overall, these accounts reflect midwives’ interpretive, experiential assessments, through which they understood vocalisations as meaningful indicators of labour progression, emotional states, and potential deviations from physiological labour. Rosa said:

The [woman’s] sound is usually adjusted to the intensity [of labour]. Especially when it comes to the progress of labour, for example, you can sometimes tell from the volume and intensity of the sound that there's progress, that we're probably getting close to the birth. (FSBC midwife Rosa)

The research participants expressed that they could easily hear and identify when birthing women had the urge to push, explaining that this is often accompanied by sounds that are distinctly different from those made during the first stage of labour. Ulrike had 28 years of experience and described the qualities of those sounds in the following way:

As if the air is held back a little. Yes, I'm just wondering if there is an instrument—like when I play the trumpet. It just happens, the pressure, this build-up of pressure, which changes something in the vocal tones; these are open, resonant tones. (Hospital and homebirth midwife Ulrike)

Several midwives described that they could hear fear. They described the sounds as high pitched, explaining that the tones made while vocal toning with fear were shorter compared to sounds that were made by birthing women who were not feeling afraid. In addition to this, several participants explained that a birthing woman might scream as a result of their fear. Ulrike described sound qualities that indicate fear to her:

Fear often comes into play [which] changes breathing and toning. [The sounds] may become higher pitched in terms of the tone, and perhaps also louder. That can happen too. (Hospital and homebirth midwife Ulrike)

The midwives shared that, based on their experience, when the baby’s head is not in an optimal position, the sounds made by the birthing woman change noticeably. The quality of the vocal expression shifts, and pain becomes audible through a qualitatively different sound. Some midwives also described that, along with the change in sound, feelings of fear could arise in the birthing woman. Marianne explained:

[Sometimes] the baby isn't in an optimal position. That's what happened with a woman who had a really big baby with her first child. And it was a high direct occiput position, and we already tried different positions and everything [to get the baby to rotate]—forward-leaning and side-lying and so on. But the baby’s head didn’t rotate. We really tried everything. But in the end, you can tell by the sounds [she makes] when the head hits the pubic bone like that. Yes, that's a different kind of pain, we know that. And you can hear it in the sounds she’s making. Sure, yes, and then it's more than that, you notice it. It's not just this toning, it's actually a cry. It's also a cry of pain that's belongs to it or is a part of it. (Homebirth midwife Marianne)

The participants elaborated that the sounds made during the second stage of labour are more distinct than those made during the first stage, where they found it more difficult to distinguish and describe the sounds. Higher-pitched sounds and screaming were an indication of emotional stress for several midwives. A non-optimal position of the baby’s head could also be indicated through the birthing woman’s vocalisations, as explained by the midwives, who noted that a quality of pain was added to the tone of the vocal expression.

### 5.3 Theme 3: Combining childbirth vocalisations, sensory perceptions and embodied cues

The midwives collected information through their sensory perceptions, which contributed to the development of embodied knowledge. This form of knowing differs from technologically mediated knowledge, such as that obtained through ultrasound examinations. Many of the midwives explained that they relied on multiple sensory modalities to form a holistic understanding of the birthing woman and their labour. This meant that, in addition to auditory perception, they also drew on visual, sensory, and, at times, olfactory cues. Before conducting further examinations or taking actions to promote the birthing woman’s well-being, the midwives emphasised that they first reflected on their perceptions of the birthing woman. In addition, some participants described using verbal inquiry and physical assessments, such as vaginal examinations, to evaluate childbirth situations in order to determine the source of pain or to assess the position of the baby’s head in relation to the pelvis, which they had become aware of through the sounds the birthing woman was making.

For combining auditory cues with other information, many midwives used visual information. Helena shared:

What comes to mind, and it happens often—is that when you hear a woman giving birth and you are outside the labour room, it sometimes sounds more alarming than when you’re actually in there yourself as a midwife. So, I think what you hear alone isn’t the deciding factor. You really have to factor in what you see as well. I think it’s difficult to judge based on hearing alone. (Hospital midwife Helena, on parental leave)

Ida first listened to the vocalisations of the birthing woman before considering whether she needed to implement a caring act. She said:

So, I think I'll try not to do too much, but rather observe and take things in before I start doing anything. Because I'm really just there to observe—in order to rule out pathology. (Hospital midwife Ida)

A holistic picture of the birthing woman emerged for Hanne as she engaged all her senses while providing care, not only through listening but also through visual cues and touch.

Once I'm in there [labour room], I can assess the situation much better, of course, because I have all the information I need, such as the position [of the woman], and, when I place my hands on her belly, I can feel how strong the contractions are. It's more challenging to assess [the sounds] from outside [of the room]. (Hospital midwife Hanne)

She further explained that visual cues were important for assessing the situation, an experience which many other midwives shared.

So, I think, based on the sounds and, other information, that I don’t need [to conduct] a vaginal examination—that I don’t actually have to check the cervix. Because I can know from other factors that she is fully dilated and pushing. So, I don't need to check her cervix in addition to that. It's not just the vocalisation—I can feel her bearing down. I can see the anus dilating. I see the purple line. So, based on the external signs, I don't care whether she has a cervical lip or not. (Hospital midwife Hanne)

During the interviews, the midwives were also asked to describe their experiences of supporting birthing women who had made little to no sounds during labour and birth. Their accounts highlighted that listening and interpreting childbirth sounds plays a central role in assessing the progress of childbirth. In addition to this, the midwives emphasised that they relied on visual and embodied cues to understand the birthing woman’s state and behaviour. Franka, who had 22 years of clinical experience, shared:

I remember she was in the tub. I can still see her in front of me. She was on all fours and kept stretching differently to make room in her pelvis. She gradually developed a reflexive urge to push. What's it called? Lilac or something like that, purple line, you could see that, too. And she also developed more secretions and whatever. You know, you could really see it. (Lecturer midwife Franka)

The midwives’ experiences of caring for women who made little or no sound during labour and birth deprived them of an important criterion for assessing both the stage and the progression of labour. Lise shared:

Lise: And at some point, she looked at me, sitting in the tub, and said: ‘When can I actually start pushing?’ And I said: ‘Well, when your body tells you to push, just do it.’ The next contraction came, she pushed, and the baby came.

Interviewer: And would you have expected the baby to come at that point?

Lise: No, I didn't even manage to put my gloves on. So, I thought, well, maybe she'll feel the urge to push soon, but she was completely introverted. (Homebirth midwife Lise)

While midwives are attuned to auditory cues, these are often intertwined with other sensory perceptions, to assess of the physiological processes of labour and birth. Visual perceptions, as well as embodied cues play an important role in the evaluation of birthing women’s vocalisations.

### 5.4 Theme 4: the multi-faceted nature of working with sound

While many midwives used vocalisations intentionally as a component during labour and birth, others did not consider vocalising to be an inherent or routine part of their practice. The interviews revealed noticeable variation in how midwives used and understood the role of vocalising. Some did not actively introduce or engage in shared vocalisation with birthing women, and therefore did not use it as a component of support. In contrast, midwives who worked intentionally with vocalisations described engaging closely with the birthing woman’s sounds to orient themselves to the progress of labour and to attune to the individual dynamics of each birth, whether by vocalising with the birthing woman, offering vocal toning tone guidance, or listening attentively. In this way, vocalisations helped the midwives to support physiological processes, including supportive breathing patterns, providing emotional support, assisting birthing women in coping with contractions, and identifying when interventions were necessary. Interestingly, rarely any midwife had thought about what effect vocalisations have on the unborn baby.

Many midwives used the birthing woman’s vocalisations as a map on which to orient themselves within the birthing woman’s labour. Tina, a hospital midwife, compared vocalisations during childbirth to a location that she could assess through listening.

Also, there are typical childbirth sounds through which I can tell where a woman is in her labour. (Hospital midwife Tina)

To support physiological progress in childbirth, Franka described using vocalisation as a non-pharmaceutical approach. She had encouraged birthing women to direct their vocal toning sounds toward the area of pain or tension, when labour was not progressing due to cervical rigidity or dystocia. She explained:

When labour starts to lose its flow, for example when I feel that it was progressing, but now she has been in pain for a while and has a tense expression on her face the whole time and she herself feels that it is not really moving forward. Especially when I was a less experienced midwife, I used to do more vaginal exams, and it was often a case of what is known as dystocia—when the cervix is tight and contracting. You could see it in her face [of the labouring woman], and I think that's absolutely the amazing key. I've experienced this with several women in labour, whom I told: Okay, now send the sound, the deep sound, exactly where the cervix hurts. Often, I mean really often, they looked totally shocked because they realised that, when they send the sound there, something happens- that they themselves can open up or that the pain eases when they send the sound there. That is always very impressive. (Lecturer midwife Franka)

Other midwives similarly highlighted engaging in shared vocal toning to provide emotional support, facilitate coping, and support physiological processes. Rosa spoke about providing companionship and stability, not only by suggesting to the birthing woman how they might meet the challenges of labour, but also by actively engaging in the process together. This helped ensure that the birthing woman did not feel alone in facing the challenges of childbirth. Rosa said:

Or when I notice, like the women [vocalising] - o, here comes another contraction peak- that’s when I try to stay constant [with my pitch], not to go up in pitch. Exactly [at that moment] I increase the volume of my voice so that she can hear me again, or at least has the chance to hear me, depending on the situation. (FSBC midwife Rosa)

Most midwives paid close attention to birthing sounds, regarding them as a meaningful source of information. Midwives elaborated that, when the birthing woman’s vocalisation changed, it served as a cue to reassess and determine whether care or an intervention was needed. These changes could include sounds indicating fear (such as a high-pitched sound), screams, expressions of bearing down, or when falling into silence. Midwives also described sounds that did not correspond to the stage of labour, for example during the placental period when they expected little or no sound. Helena explained:

When the tone suddenly changes, so if the woman is vocalising a single, monotone tone the whole time, always using a similar tone, then I know that everything is probably fine. But if a new tone suddenly comes in, then I take a closer look. (Hospital midwife Helena, on parental leave)

For the midwives, specific tones and qualities of the vocalisation signalled if an intervention was necessary. The birthing woman's vocalisations showed the midwives that she was managing the childbirth well and that it was progressing within physiological norms. Maren described how she used her perception to assess the sound quality in order to identify a potential need for action:

Well, if the woman is loud, feels good and needs to be or is powerful. Um, then I don’t feel like I have to do anything. (Hospital and FSBC midwife Maren)

Several midwives shared approaches they had personally developed for working with vocal toning and other vocalisations during pregnancy, labour and birth, such as overtone singing. Gundi, who demonstrated this technique during her interview, modulated the overtone by shaping the tongue and mouth cavity, producing a deep, dark tonal quality. Yet another example was given by Marianne, who incorporated a G-major triad into her practice. Through this musical structure, she created an accessible, low-threshold way for birthing women to engage in vocalisation during childbirth. Because singing is familiar and socially acceptable, it was considered less likely to evoke feelings of shame associated with internalised social norms. She introduced triad toning during antenatal courses and sang with participants both in antenatal care and birth preparation courses to help pregnant women become comfortable with their own voices. She demonstrated her work with sounds during the interview:

Very long tones: Sa Ba Ja Sa (IPA: [zɑ: bɑ: jɑ: zɑ:]) ((Sings G-major triad: G–H-D-G)). (Homebirth midwife Marianne)

Participants also described creative vocal practices initiated by the birthing women themselves. One midwife, for example, demonstrated a birthing woman’s airy, breath-infused singing during contractions. The midwife demonstrated this: "Tochter ((takes a breath)) Zi-on. ((takes a breath)) Freu-he-he-he dich", IPA: [tɔx.tɐ.hɐ . tsi:.ɔn . fRɔɪ.hə.hə.hə.hə.hə . dɪç] ((with a lot of air, the dots represent the syllable boundaries)). Another example involved yodeling. Marianne demonstrated the rapid alternation between head and chest voice typical of this style, IPA: [jɛ hy: hi: hɔl dejɛ hy: hi:].

These examples illustrate that vocalisation is meaningful for midwifery practice, functioning not only as a component of care to support physiological processes during childbirth, but also as a means of attunement and mutual preparation during pregnancy between midwives and birthing women. The midwives employed vocalisations in various ways to promote well-being and support the physiological processes of each birthing woman in their care.

## 6 Discussion

The results of this study show that midwives work with birthing women’s vocalisations as part of a broader, multisensory assessment and promotion of physiology and well-being during childbirth. Midwives use vocalisations to assess labour progress and dynamics, identify deviations from physiology, guide care decisions, and support coping with the pain and intensity of contractions. In addition, some midwives use vocalisations as a clinical tool to facilitate emotional regulation, and to support the establishment of effective breathing patterns. Auditory perception, rather than just a passive reception of sound, was described as an interpretive and relational process through which meaning is constructed. For these midwives, attuned listening constituted an embodied form of knowing, developed through experience and attunement to the birthing woman rather than through formal instruction alone. Taken together, the four themes suggest an epistemic process through which midwives develop embodied clinical knowledge in practice. This process involves learning to listen, recognising nuanced sound patterns, integrating auditory cues with other sensory and embodied information, and developing the acquired knowledge further in a variety of ways to apply it individually in their care during childbirth.

A key finding of this study was that vocalisations were rarely interpreted in isolation. Rather, they were understood as a part of a broader, multisensory process. This multisensory integration represents a key stage in the development of embodied clinical knowledge, as midwives move from recognising isolated cues to interpreting complex, interrelated signals in practice. Midwives in this study consistently described integrating what they heard with visual cues, bodily signals, and tactile information to form a holistic understanding of the birthing woman and the labour process (Davis-Floyd, 2018; Stone et al., 2023). This aligns with broader understandings of perception as an active, multisensory process, in which meaning arises through the coordination of different perceptual systems (Gibson, 1966). In childbirth settings where midwives are responsible for more than one birthing woman, surveillance techniques frequently form the basis for clinical assessment, including continuous fetal heart monitoring, intrapartum ultrasound, and vaginal examinations, while perceptions and embodied experiences of the birthing woman are neglected or discounted (Nilsson et al., 2019; Newnham et al., 2017; Moncrieff et al., 2022; Hinkson et al., 2025; Stone et al., 2023). The findings of this study highlight auditory perception as a clinically meaningful source of information that supports ongoing assessment, and decision-making. Moreover, auditory attunement to vocalisations plays an important role in supporting physiological childbirth, as it allows midwives to recognise, respond to, and, when appropriate, guide vocal expression during childbirth based on their experiential assessment (McKay and Roberts, 1990; Pierce, 1998; Dahan and Goldberg, 2025).

From a nursing and midwifery perspective auditory attunement reflects an embodied way of gaining knowledge about a birthing woman. Rather than treating the body solely as an object of measurement (Stone et al., 2023; Walsh, 2006), midwives described working with vocalisations as a way to engage with the birthing woman’s body as lived, expressive, and relational. Through auditory perception, midwives’ knowledge acquisition involved interpretation and relational understanding, rather than sensory registration alone (Merleau-Ponty, 1962). In this sense, knowledge is generated through close sensory engagement with the woman in labour, integrating bodily signs, vocal expressions, and emotional presence (Engelhart et al., 2022; Reis da Silva, 2024). This understanding resonates with nursing scholarship that conceptualizes clinical knowledge as arising from the integration of the physical body with lived experience, positioning perception as central to professional judgement and care (Sandelowski, 2000; Lawler, 1991). Lawler (1991) wrote that the nurse’s knowledge of a patient is obtained from the body, a concept she called somology. Somology is “*understanding the body as an integration of the object body (the thing) into experience so that it is simultaneously an object, a means of experience, a means of expression, a manner of presence among other people, and a part of one’s personal identity.*” (Lawler, 1991, p. 29). Consequently, integrating auditory perceptions into care of the birthing woman draws the attention of the midwife to an added layer of clinical information, bringing the embodied experience of the birthing woman more clearly into view.

When midwives have developed skilled listening, they are able to perceive specific and differentiated aspects of childbirth through sound. As this process develops, midwives’ auditory perception becomes more refined, enabling increasingly nuanced interpretations of vocalisations in relation to labour progression. The ability to discern nuances in vocalisations ranged from basic auditory distinctions, such as identifying different stages of labour, to more advanced perceptions, including midwives’ interpretive understandings of sounds in relation to fetal malposition. Previous research has shown that midwives are able to differentiate between vocalisations associated with different phases of labour (Baker and Kenner, 1993; McKay and Roberts, 1990), and our findings suggest that sounds produced during the second stage of labour may be perceived as more consistent and clearer than those of the first stage. The midwives interviewed in this study expressed particular confidence and ease in naming and describing vocalisations associated with the urge to push, indicating a well-established auditory familiarity with this phase of labour. Existing research from a midwifery perspective has demonstrated that midwives are able to perceive emotional states, such as fear, through birthing women’s vocalisations (McKay and Roberts, 1990; Pereira et al., 2025), which is consistent with our findings. However, to our knowledge, previous studies have not described auditory perceptions related to fetal malposition. The ability described by some midwives in this study to hear indications of malposition, therefore, represents a novel contribution and highlights the need for further research into sensory-based ways of identifying physiological and pathological processes in childbirth.

Unlike within the context of illness, where vocal expressions of pain are typically interpreted as signs of pathology, the sounds birthing women make during labour were understood by midwives in this study as potentially signaling physiological, and well-being, as well as deviations from this. This pattern of learning reflects the progressive development of embodied knowledge, in which midwives move from guided interpretation toward intuitive and context-responsive clinical judgement. For birthing women, feedback from midwives has been shown to support coping and vocal regulation (Zinsser and Engebakken Flaathen, 2026; Pierce, 1998). In our study, midwives developed their auditory perceptual skills through years of clinical practice. This learning process was shaped, in particular, by continuous one-to-one care during labour and birth, which allowed midwives to hear and listen closely to the soundscape of both the physical and emotional aspects of childbirth (Revision, 2026). This pattern of learning suggests that auditory perception develops as a form of situated knowledge, emerging through practice, action, and reflection within the clinical context (Ataizi, 2012; Lave and Wenger, 1991). Rather than being transmitted primarily through formal instruction, this knowledge is constructed through embodied participation in care and through relational learning with more experienced practitioners (Stone et al., 2024). Externally guided interpretations, such as those offered by colleagues or mentors, became internalised, enabling midwives to respond tacitly and confidently to what they heard (Polyani, 1967). This process reflects a form of experiential apprenticeship, in which auditory perception is cultivated through observation, shared sense-making, and reflection. The relative absence of structured education on vocalisations within midwifery training therefore highlights a gap between formal curricula and the embodied skills required in clinical practice. This is underpinned by studies (Roosevelt et al., 2025; Pereira et al., 2025) that shared similar findings about the lack of training for midwives to work with vocalisations during childbirth.

Taken together, these findings indicate that vocalisation functions as a clinical and relational component of care within midwifery practice, encompassing a range of meanings and uses aimed at supporting both well-being and physiological processes during childbirth.

### 6.1 Implications for practice

This study suggests that auditory perception is a meaningful, though often implicit, component of midwifery practice during labour and birth. Greater awareness of vocalisations as a source of relational information may support midwives in assessing and promoting physiological progress, emotional well-being, and the personalisation of supportive care. Introducing foundational concepts related to childbirth vocalisations within midwifery education, alongside opportunities for reflective learning and mentorship, would help make this embodied knowledge more explicit and transferable within the profession. In situations where pregnant women need additional support, midwives may consider working with vocalisations as an initial caring response. This may include supporting emotional well-being; facilitating physiological progress (e.g. in cases of cervical dystocia); assisting with coping with pain; establishing supportive breathing patterns; and supporting the birthing women’s sense of connection to their baby and their embodied engagement during contractions, including when epidural analgesia is used.

### 6.2 Strengths and limitations

To ensure a diversity of experience, this study included midwives from different regions of Germany with diverse lengths of professional experience and practice settings, which may support the transferability of the findings. However, transferability may be limited in contexts where midwives are the primary care providers for childbirth, such as in some healthcare systems outside of Europe. Several interviews were conducted via video conference and telephone, which may have influenced the interview process, as nonverbal cues could not always be observed. At the same time, these formats enabled the inclusion of midwives from a wide geographical area, as well as participants who were not very familiar with digital technology. Methodological rigour was ensured through adherence to the criteria for trustworthiness outlined by Lincoln and Guba (1985), and through transparent reporting in line with the COREQ checklist (Tong et al., 2007). Given the cultural, social, and individual variation in vocalisation during childbirth (Aziato et al., 2017; Danford et al., 2022; Zinsser and Stone, 2025), both the interpretation and clinical relevance of vocalisations may vary. Accordingly, the transferability of these findings is likely greatest in contexts where vocal self-expression during labour is accepted and encouraged.

## 7 Conclusion

This study highlights how understandings of vocalisations during childbirth are perceived and shaped through midwifery practice and relational care. Auditory attunement to vocalisations functions as a meaningful component of care of birthing women, contributing to the assessment of physiological processes and emotional well-being. In addition, vocalisation may be used antenatally as a supportive component of care to help prepare for labour.

Midwives integrated auditory perception with other sensory and embodied cues to form holistic clinical judgements. Although approaches to listening varied, auditory perception consistently informed midwives’ understanding of the labour process and their responses to birthing women. Integrating auditory perception into midwifery education may help to strengthen practice-based skills and support non-technological approaches to caring for birthing women who desire a physiological childbirth.

## Declaration of generative AI and AI-Assisted technologies in the writing process

No AI was used.

## Data availability

Interview data are not publicly available for ethical reasons. Processed materials are available from the corresponding author upon reasonable request.

## CRediT authorship contribution statement

**Laura A. Zinsser:** Writing – review & editing, Writing – original draft, Visualization, Validation, Project administration, Methodology, Investigation, Conceptualization. **Nancy I. Stone:** Writing – review & editing, Writing – original draft, Validation.

## Declaration of competing interest

The authors declare that they have no known competing financial interests or personal relationships that could have appeared to influence the work reported in this paper.
